# Cervical Myelopathy and Social Media: Mixed Methods Analysis

**DOI:** 10.2196/42097

**Published:** 2023-05-22

**Authors:** Lior M Elkaim, Jordan J Levett, Farbod Niazi, Mohammed A Alvi, Nathan A Shlobin, Joseph R Linzey, Faith Robertson, Rakan Bokhari, Naif M Alotaibi, Oliver Lasry

**Affiliations:** 1 Department of Neurology and Neurosurgery McGill University Montreal, QC Canada; 2 Department of Medicine University of Montreal Montreal, QC Canada; 3 Division of Neurosurgery Department of Surgery University of Toronto Toronto, ON Canada; 4 Department of Neurological Surgery Feinberg School of Medicine Northwestern University Chicago, IL United States; 5 Department of Neurosurgery University of Michigan Detroit, MI United States; 6 Department of Neurosurgery Massachusetts General Hospital Harvard Medical School Boston, MA United States; 7 National Neuroscience Institute King Fahad Medical City Riyadh Saudi Arabia

**Keywords:** social media, twitter, cervical, myelopathy, spine, neurological, condition, degenerative, patient, caretaker, clinician, researcher, user, tweets, engagement, online, education, support

## Abstract

**Background:**

Degenerative cervical myelopathy (DCM) is a progressive neurologic condition caused by age-related degeneration of the cervical spine. Social media has become a crucial part of many patients’ lives; however, little is known about social media use pertaining to DCM.

**Objective:**

This manuscript describes the landscape of social media use and DCM in patients, caretakers, clinicians, and researchers.

**Methods:**

A comprehensive search of the entire Twitter application programing interface database from inception to March 2022 was performed to identify all tweets about cervical myelopathy. Data on Twitter users included geographic location, number of followers, and number of tweets. The number of tweet likes, retweets, quotes, and total engagement were collected. Tweets were also categorized based on their underlying themes. Mentions pertaining to past or upcoming surgical procedures were recorded. A natural language processing algorithm was used to assign a polarity score, subjectivity score, and analysis label to each tweet for sentiment analysis.

**Results:**

Overall, 1859 unique tweets from 1769 accounts met the inclusion criteria. The highest frequency of tweets was seen in 2018 and 2019, and tweets decreased significantly in 2020 and 2021. Most (888/1769, 50.2%) of the tweets’ authors were from the United States, United Kingdom, or Canada. Account categorization showed that 668 of 1769 (37.8%) users discussing DCM on Twitter were medical doctors or researchers, 415 of 1769 (23.5%) were patients or caregivers, and 201 of 1769 (11.4%) were news media outlets. The 1859 tweets most often discussed research (n=761, 40.9%), followed by spreading awareness or informing the public on DCM (n=559, 30.1%). Tweets describing personal patient perspectives on living with DCM were seen in 296 (15.9%) posts, with 65 (24%) of these discussing upcoming or past surgical experiences. Few tweets were related to advertising (n=31, 1.7%) or fundraising (n=7, 0.4%). A total of 930 (50%) tweets included a link, 260 (14%) included media (ie, photos or videos), and 595 (32%) included a hashtag. Overall, 847 of the 1859 tweets (45.6%) were classified as neutral, 717 (38.6%) as positive, and 295 (15.9%) as negative.

**Conclusions:**

When categorized thematically, most tweets were related to research, followed by spreading awareness or informing the public on DCM. Almost 25% (65/296) of tweets describing patients’ personal experiences with DCM discussed past or upcoming surgical interventions. Few posts pertained to advertising or fundraising. These data can help identify areas for improvement of public awareness online, particularly regarding education, support, and fundraising.

## Introduction

Degenerative cervical myelopathy (DCM) [1] is the most common cause of spinal cord dysfunction in the world [2]. Results from epidemiological studies on DCM vary widely and may underestimate true disease prevalence for DCM; however, current estimates place DCM incidence and prevalence in North America at a minimum of 41 and 605 per million, respectively [3]. DCM is a disease that is often poorly understood by the public, and, at times, by nonsurgical clinicians [4]. This leads to significant diagnostic delays that, along with the progressive neurologic dysfunction seen in DCM, cause major individual disease burden [5]. From a societal perspective, the current best available data estimates that DCM has an annual cost of over £681 million (US $845 million) per year in the United Kingdom, mainly due to admissions costs, lost productivity, and disability payments [6]. For individuals with myelopathy, experiences can vary greatly. Several activities of daily life can be affected, including walking, toileting, and dressing [7].

Studies analyzing the perspectives of individuals with various diseases, including DCM, have become increasingly important to better understand the treatment objectives of patients and their caretakers [8-10]. However, there is little research on the effects of DCM from the perspectives of patients. To our knowledge, there exists only one study examining outcomes of DCM through the lens of patients living with the condition. The study, by Davies and colleagues [11], used an online survey of DCM patients to identify symptoms and handicaps caused by the illness. Otherwise, little is known about patient and caretaker perspectives on DCM [9,10]. One of the best ways to understand these perspectives is through social media, where patients often post about living with their disease [12].

Social media has become a powerful tool, shaping modern commercial and political discourse. With over 190 million active daily users on Twitter [13], social media has become indispensable to some patients, caregivers, clinicians, and researchers. Patients use social media for several reasons, including to ask for encouragement, to discuss novel treatment strategies, and to raise awareness of their illness [14]. Clinicians and researchers use social media to facilitate communication with patients, fundraise, and advertise services and medical centers [15]. Neurosurgical journals, like the Journal of Neurosurgery (JNS), now have specialized social media teams dedicated to improving targeted digital outreach [16].

Further, due in part to the large public presence on Twitter and Twitter’s suitability for data mining, researchers have leveraged social media to gather insight on public opinion, knowledge transfer, and patient perspectives [17]. This has led to publications on social media use related to several neurosurgical pathologies. A 2021 systematic review found 29 peer-reviewed publications related to social media use in neurosurgery, with most posts relating to requests or providing information and seeking emotional support or forming connections [18]. Despite this, there has not yet been a study analyzing social media use as it pertains to DCM. This paper aims to contribute to the social media and neurosurgery literature by describing the landscape of social media use related to DCM. Lessons gleaned from this analysis include patient perspectives and sentiment and evolution of social media use over time. Further, social media research can help clinicians understand the online space, leading to improved interactions with patients and optimized education, support, and treatment plans.

## Methods

### Search Strategy

A comprehensive search of the Twitter database for academic research was performed in March 2022. We attempted to extract all tweets pertaining to DCM in humans, without restriction. The following keywords were used for the search: *cervical myelopathy* or *cervical* [AND] *myelopathy*. The methods in this study were based on methods described in previous studies [15,19]. We analyzed tweets and accounts individually.

### Accounts

The following types of accounts were excluded: (1) duplicate accounts, (2) accounts with less than 10 tweets, (3) bots, and (4) accounts with less than 15 followers. Accounts were categorized as bots if their usernames identified them as such or if all tweets on the account were retweets using identical formatting. The following data were extracted for further analysis: account location, number of followers, number of tweets, and year joined. Account categories were created based on manual screening of categories derived from previous publications [15]. The created categories were based on interpretation of the accounts’ purpose, defined by their tweets, usernames, and account descriptions. Accounts were categorized by 2 independent reviewers with content expertise (FN and JJL), while a third (LME) verified accuracy. The final account categories were *foundation*, *business*, *journal*, *patient/caregiver, private citizen*, *support group*, *medical center*, *news outlet*, *medical doctor/researcher*, and *other*.

### Tweets

Individual tweets (ie, posts) were selected as the main subject of analysis in this paper. Analysis of individual posts, rather than analysis of account descriptions, likely represents the most meaningful method by which the social media landscape for a specific pathology can be defined [20]. Several data points were extracted for each individual tweet, including tweet date, number of likes, retweets, quote tweets, replies, full tweet text, presence of media, presence of hashtags, and presence of tagging. All duplicates were removed, and only tweets relevant to DCM in humans were included for analysis. This was defined by a manual read through of all included tweets. All included tweets were categorized into different groups based on modified thematic analysis further described in other papers on social media analysis [15]. Tweet categorization was done independently by 2 reviewers (LME and JJL) with differences resolved through discussion. The final tweet categories were *personal experiences*, *raising awareness*, *discussing research or publications*, *advertising*, *fundraising*, and *other*. A word-cloud composed of words derived from included tweets can be found in Multimedia Appendix 1.

### Statistical Analysis

In line with previous studies, we used descriptive statistics (median, IQR) for the following social media metrics: followers, tweet count, tweet likes, retweets, and quote tweets. Total tweet engagement, including a sum of tweet likes, retweets, and quote tweets, was also calculated. R (version 4.1.3; R Foundation for Statistical Computing) was used for all statistical analyses.

### Sentiment Analysis

To determine negative or positive outlook for tweets (ie, their sentiment), we used a natural language processing (NLP) python library, *TextBlob* [21]. *TextBlob* works by assigning a score to the tweet polarity and subjectivity. These scores are calculated using a predefined dictionary of words that analyzes data semantically. The polarity score is used to represent sentiment, with –1 representing the most negative tweets and +1 representing the most positive tweets. The subjectivity score (range 0-1) is used to represent subjectivity, with 0 being objective and 1 being subjective (or personal). Finally, the algorithm assigns an analysis label, defined as follows: scores of less than 0 represent negative sentiment, scores of 0 represent neutral sentiment, and scores of greater than 0 represent positive sentiment.

### Ethical Considerations

This study is compliant with the Canadian Tri-Council Policy Statement for Research, which stipulates that research conducted with data that are publicly available does not require formal institutional research ethics board approval [22]. This is because all included tweets in this study were obtained from a publicly accessible source (Twitter) using data that were voluntarily posted by public accounts to a public forum. No tweets from private (ie, locked) Twitter accounts were included, and all usernames were omitted from analysis.

## Results

### Overview

A complete search of the Twitter application programming interface database from inception to March 2022 yielded 6262 tweets. Of these, 1859 tweets from 1769 accounts were kept for analysis after duplicate tweets, retweets, tweets from bots, and nonsensical tweets were removed.

### Account Analysis

The median number of followers for the accounts was 722 (IQR 183-2547). The mean number of followers for all accounts was 5423 (SD 51421). The median number of tweets per account was 4543 (IQR 1133-19,095). Overall, most of the 1769 accounts were from English-speaking countries, including the United States (n=533, 30.2%), the United Kingdom (n=264, 15%), and Canada (n=91, 5.1%). The 3 non–English-speaking countries with the highest number of tweets were India (n=44, 2.5%), Spain (n=26, 1.5%), and Saudi Arabia (n=18, 1%). Almost 30% (n=540) of accounts did not have a listed location. Accounts were categorized as *medical doctor/researcher* in 668 of 1769 (37.8%) cases, *patient/caregiver* in 415 (23.5%), *news media outlet* in 201 (11.4%), *medical center* in 166 (9.4%), and *foundation* in 74 (4.2%). There were relatively few tweets from businesses (n=62, 3.5%), medical journals (n=55, 3.1%), and support groups (n=23, 1.3%). A breakdown of account data, including classification and location, can be found in Table 1.

**Table 1:** Characteristics of accounts included in the final analysis (n=1769).

Characteristics		Values
Followers, median (IQR)		722 (183-2547)
Number of tweets per account, median (IQR)		4543 (1133-19,095)
**Category, n (%)**		
	Medical doctor/researcher	668 (37.8)
	Patient/caregiver	415 (23.5)
	News	201 (11.4)
	Foundation	74 (4.2)
	Business	62 (3.5)
	Journal	55 (3.1)
	Support group	23 (1.3)
	Other	104 (5.9)
**Account location, n (%)**		
	United States	533 (30.2)
	United Kingdom	264 (15)
	Canada	91 (5.1)
	India	44 (2.5)
	Spain	26 (1.5)
	None	540 (30.5)
	Other	271 (15.3)

### Tweet Analysis

Overall, 1859 unique tweets meeting the inclusion criteria were extracted for analysis. Less than 100 tweets per year were seen from 2010 to 2014. The highest frequency of tweets related to DCM was seen in 2018 and 2019, with 305 (16.4%) and 311 (16.7%), respectively. After 2019, tweet counts dipped to 161 (8.7%) in 2020 and to a low of 110 (5.9%) in 2021.

The median engagement count (including all likes, replies, retweets, and quotes) per tweet was 1 (IQR 0-3), while the mean engagement count was 4 (SD 15.5). The mean number of likes and retweets per tweet was 2.1 (SD 10.0) and 1.7 (SD 8.6), respectively. A total of 930 (50%) tweets included a link, 260 (14%) included media (ie, photos or videos), and 595 (32%) included a hashtag. Sentiment analysis classified 847 (45.6%) of the tweets as neutral, 717 (38.6%) as positive, and 295 (15.9%) as negative. An overview of tweet characteristics, including tweet categories, can be found in Table 2.

**Table 2:** Characteristics of included tweets (n=1859).

Characteristics			Values
**Category, n (%)**			
	Advertising	31 (1.7)	
	Awareness	559 (30.1)	
	Experience	296 (15.9)	
	Fundraising	7 (0.4)	
	Other	205 (11)	
	Research	761 (40.9)	
Total engagement, median (IQR)			1 (0-3)
Total engagement, mean (SD)			4.1 (15.5)
**Engagement by category, mean (SD)**			
	Replies	1.75 (8.59)	
	Retweets	0.15 (0.84)	
	Likes	2.14 (10.03)	
	Quotes	0.07 (0.40)	
**Sentiment, n (%)**			
	Positive	717 (38.6)	
	Negative	295 (15.9)	
	Neutral	847 (45.6)	

### Tweet Themes

#### Research

*Research* was the most common (761/1859, 40.9%) theme. Often, these tweets were about new publications or treatments. For example, one medical doctor tweeted, “The study authors wrote that surgical treatment ‘cannot only arrest further progression of myelopathy but also improve functional status, neurological outcomes, and quality of life’ [link omitted].” Further, tweets in this group were often made by surgical journals, including the following from the JNS: “#OnlineFirst: Social risk factors predicting outcomes of cervical myelopathy surgery [link omitted].”

#### Awareness

Tweets included in this category focused on spreading DCM awareness or informing readers about DCM. This was the second most common tweet theme, seen in 30.1% (559/1859) of cases. Often, these tweets sought to inform the public on DCM, including through the presentation of general information, such as “#Cervical myelopathy is the most common #spinal cord disorder in older adults. Learn more here: [link omitted]” and “Cervical myelopathy is estimated to affect up to 5% of people older than 40 years, and incidence is expected to rise in ageing populations.”

#### Personal Experience

Tweets about personal experiences with DCM were seen in almost 16% (296/1859) of posts. These tweets were often, but not always, made by individuals with cervical myelopathy. For example, one user tweeted, “My disability is neurological as well - cervical myelopathy with klippel feil anomalies - very painful but I'm awaiting an operation soon which I hope will make things a little easier,” while another tweeted “When my condition, cervical myelopathy started getting worse again. I turned back to music as something that has always been my ‘rock.’” Overall, of the 296 tweets in the personal experiences category, 59 (24%) discussed upcoming or previous surgical interventions. Examples include “Kindly Pray for my Father he is going to have cervical myelopathy surgery very critical operation,” “My surgery was a [discectomy]. They went in through my throat. My condition was cervical myelopathy. Not good. Google it,” and “[tag] Did I tell you surgery for my cervical myelopathy is 11/21...”

#### Advertising and Fundraising

There were relatively few tweets in the advertising or fundraising groups. Overall, 31 of 1859 (1.7%) tweets were advertisements, while only 7 of 1859 tweets (0.4%) concerned fundraising. All 7 tweets related to fundraising were appeals by private citizens for financial support for individuals with cervical myelopathy, and none were from registered charities. Tweets in the advertising group usually discussed upcoming educational opportunities, including workshops and seminars, or appealed to the scientific community to fill surveys for research. Tweets that did not fall into any category, such as those replying to Twitter polls or case examples, were included in the *other* category.

## Discussion

### Principal Findings

To the best of our knowledge, this is the first study to describe social media use and DCM. Several findings are reported, including that (1) most tweets were from medical doctors or researchers, (2) tweet subject matter usually related to novel research findings, spreading awareness of DCM, or personal patient experiences, (3) almost a quarter of tweets in the *personal experiences* category discussed upcoming or past surgery for DCM, and (4) most tweets were classified as positive or neutral in a sentiment analysis.

### Tweet Themes

Posts discussing research, spreading awareness of DCM, and discussing personal experiences of patients or caregivers accounted for over 85% (1589/1859) of the tweets included in this study. Although social media analysis has been performed for several neurosurgical pathologies, including epilepsy [15], aneurysms [19], and hydrocephalus [23], tweet categorization has often identified different themes from those found in our analysis. For example, a study on social media and epilepsy identified *providing information*, *providing support*, and *advertisement* as the most encountered post categories [15]. Another study on selective dorsal rhizotomy in patients with cerebral palsy showed that 31.9% of Facebook, YouTube, and Twitter comments were for *appreciation and successes*, 22.3% for *emotional support*, and 16% for *sharing information and advice* [24]. Although there is some overlap in themes in different social media studies, the lack of categorical consistency limits the ability to compare results between studies. Future social media analyses should, when possible, seek to use the same or similar categories as those that have been previously published.

Most (n=761, 40.9%) of the tweets included in the analysis were related to research. This result is consistent with data from our group on social media use related to pediatric deep brain stimulation (DBS), in which 45% of tweets fell under the *research* category [25]. Very few tweets were related to advertisement or fundraising. The low number of tweets related to fundraising is in keeping with the low general awareness and public interest in DCM. At the time of the writing of this paper, there is only one registered charity, Myelopathy.org, dedicated to DCM. Improving awareness of DCM has recently been named as the number one research priority identified by the AO Spine Research Objectives and Common Data Elements for Degenerative Cervical Myelopathy (RECODE-DCM) initiative [26]. In the future, analyzing tweets related to DCM charities and fundraisers could be an inexpensive way to track progress on AO Spine’s goal.

Notably, 269 of 1859 (16%) tweets were categorized as personal experiences in dealing with DCM, either as a patient or caretaker. Of these 269 tweets, 65 (24%) directly discussed an upcoming or past surgical procedure. Generally, studies on social media in neurosurgery do not specify how many posts mention surgical intervention. Comparatively, only 1% of posts mentioned surgical interventions in a 2017 study on social media and epilepsy [15]. Overall, the proportion of people or caregivers tweeting about DCM reflects the potentially important role social media platforms play in their lives. Social media can serve as an outlet, allowing patients to acquire or share information, seek or provide emotional support, discuss personal triumphs or failures, and mourn or express grief [14,27,28]. Social media has become so important for some that it has been described as a form of “vital media” in young patients with cancer, which is characterized by (1) actively using social media to generate a sense of well-being or balance, (2) experiencing social media as a vital technology that can sometimes expose an individual to unpredictable content, and (3) sharing important milestones of illness, including positive experiences rather than negative experiences [28]. Over the next several years, social media use is expected to continue to rise [29], especially among younger generations. It is therefore increasingly important for clinicians to understand these platforms and to optimize communication networks between clinical stakeholders and patients. As more data emerge, comparisons of social media use in DCM and in other pathologies with a similar epidemiology (ie, multiple sclerosis) will be possible [30].

### Tweet Timeline

A steady increase in tweets per calendar year was seen from 2010 to 2016, followed by a small decline in 2017 and a peak in 2018 and 2019. Tweet counts subsequently decreased significantly in 2020 and 2021. The tweet count obtained from 3 months in 2022 (January to March) was higher than the entire tweet count in 2020 or 2021. The drop in tweets is somewhat unexpected, given a net 11% increase in Twitter users year over year in 2021 compared to 2020 [31]. It could perhaps be due to increased public focus on COVID-19, with less attention given to other diseases [32,33]. The significant increase in tweets related to DCM in 2022 could reflect a shift in public attention back from COVID-19 to certain chronic pathologies.

### Sentiment Analysis

NLP for sentiment analysis has been used in several political [34], commercial [35], and medical studies [36]. Sentiment analysis aims to identify positive and negative opinions and emotions expressed in free-text natural language [37]. In this way, researchers can determine the feelings social media users have toward certain topics, or in medicine, certain treatments. In health care, sentiment analysis has been used to determine the outlook of Twitter users toward several subjects, including e-cigarettes [38], tobacco use [39], palliative medicine [40], and multiple sclerosis treatments [41]. However, formal sentiment analysis using NLP has rarely been used in studies on social media related to neurosurgical pathologies [42]. In our study on DBS in children, most tweets were either positive or neutral (55% and 35%, respectively), with only 10% having a negative outlook. Comparatively, tweets related to DCM included in this study were more likely to be neutral or negative (46% and 16%, respectively).

### Strengths and Limitations

The strengths of this study include (1) being the first to analyze social media use related to DCM, (2) being comprehensive and capturing all tweets related to DCM that met rigorous and reproducible inclusion criteria, (3) using both qualitative and quantitative methods to analyze data, and (4) using NLP on the included tweets to identify user sentiment. The efficiency and accuracy of NLP continue to improve as refinements to input features, classifiers, and models are made [43].

This study also has limitations. The data included were derived directly from Twitter and did not capture patient perspectives on other social media platforms like Facebook, YouTube, Instagram, or Reddit. The perspectives on these other social media platforms may differ from those on Twitter, though this has not been systematically investigated. Further, the demographics of social media users likely differ by platform. It is possible that more younger users are on Twitter, while an older demographic uses Facebook. Thus, a study analyzing perspectives on DCM on other social media platforms is warranted. It is also possible that some tweets were made by bots, although great care was taken to exclude these through manual categorization of each tweet. Further, DCM is a global disease and affects people in different ways across the world. It is therefore important to recognize the diversity in perspectives regarding the measurement of disability for DCM. Finally, it is possible that tweets related to cervical myelopathy were missed by the search strategy; “cervical myelopathy” is a medical diagnosis, and many patients and caregivers may refer to the pathology using other terms, such as “compressed cord” or “neck pain.” However, these terms were not included in the search strategy to increase specificity for tweets directly related to DCM.

### Conclusion

Medical doctors, researchers, and patients or caregivers are the most active groups on Twitter. When categorized, most tweets related to research, spreading awareness, or patient perspectives. Almost 25% (65/269) of tweets describing personal patient experiences discussed past or upcoming surgery. Sentiment analysis shows that roughly 16% (295/1859) of tweets had a negative outlook on DCM. There were relatively few posts related to advertising or fundraising. These analyses identified several gaps for social media use in DCM, particularly regarding spreading awareness and fundraising. The data can also be used to help clinical stakeholders better understand the increasing role social media plays in many patients’ lives.

## Appendix

Multimedia Appendix 1 Word cloud generated using all included Tweets. Font size correlates with usage, where larger words are more common.

## Abbreviations

DBS: deep brain stimulation
DCM: degenerative cervical myelopathy
JNS: Journal of Neurosurgery
NLP: natural language processing
RECODE-DCM: Research Objectives and Common Data Elements for Degenerative Cervical Myelopathy

## Data Availability

The data sets generated during and/or analyzed during this study are available from the corresponding author on reasonable request.
