# Supplementary figures and images for: Cervical Myelopathy and Social Media: Mixed Methods Analysis
Source: J Med Internet Res. 2023 May 22;25:e42097. doi: 10.2196/42097 (PMC10242472; doi:10.2196/42097)

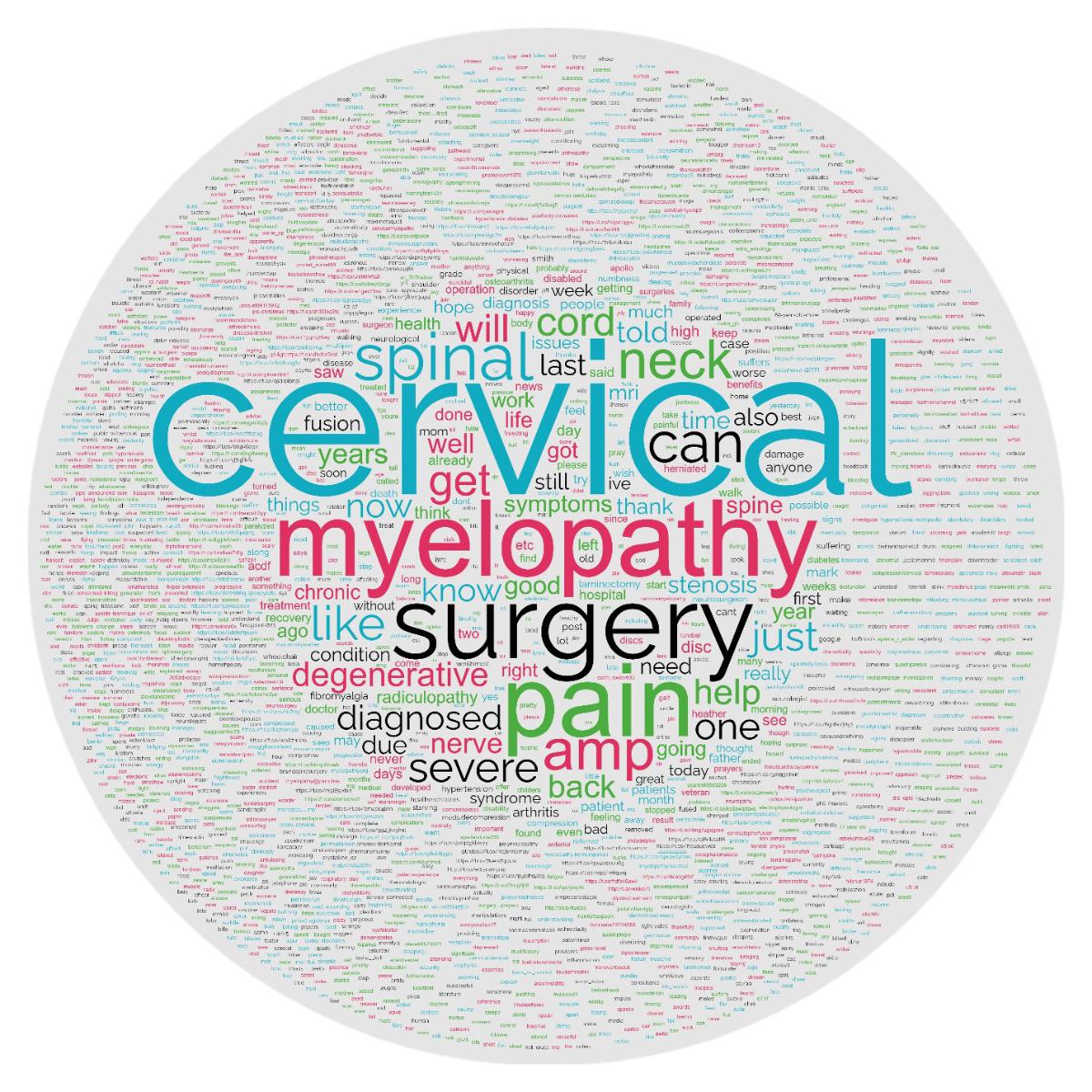

Supplement: Multimedia Appendix 1 [file jmir_v25i1e42097_app1.png]
